# Suicide Prevention and Follow-Up Services: A Narrative Review

**DOI:** 10.5539/gjhs.v8n5p145

**Published:** 2015-09-28

**Authors:** Behrooz Ghanbari, Seyed Kazem Malakouti, Marzieh Nojomi, Kaveh Alavi, Shiva Khaleghparast

**Affiliations:** 1 Mental Health Research Center (MHRC), Tehran Institute of Psychiatry, Faculty of behavioral sciences and mental health, Iran University of Medical Sciences (IUMS), Tehran, Iran; 2 Department of Community Medicine, School of Medicine, Iran University of Medical Sciences (IUMS), Tehran, Iran; 3 Center for Nursing Care Research (CNCR), Iran University of Medical Sciences (IUMS), Tehran, Iran

**Keywords:** suicide attempt, suicide prevention, follow-up

## Abstract

Previous suicide attempt is the most important predictor of death by suicide. Thus preventive interventions after attempting to suicide is essential to prevent reattempts. This paper attempts to determine whether phone preventive interventions or other vehicles (postal cards, email and case management) are effective in reattempt prevention and health promotion after discharge by providing an overview of studies on suicide reattempts. The research investigated in this review conducted from 1995 to 2014. A total of 26 cases related to the aim of this research were derived from 36 English articles with the aforementioned keywords Research shows that providing comprehensive aids, social support, and follow-up after discharge can significantly prevent suicide reattempts. Several studies showed that follow-up support (phone calls, crisis cards, mails, postal cards.) after discharge can significantly decrease the risk of suicide. More randomized controlled trials (RCT) are required to determine what factors of follow-up are more effective than other methods.

## 1. Introduction

### 1.1 Background

Suicide is a global challenge and a large concern for public health across the world (Yip, 2011). Suicide is the tenth leading cause of death and the third leading cause of death among individual from 15 to 34 (Hawton & van Heeringen, 2009). Around 800 000 to 1 000 000 people (one every 40 seconds) die by suicide each year (World Health Organization, 2014). The World Health Organization estimates that about 1.53 million people will die by suicide in 2020 (World Health Organization, 2004). Some 73% of Suicides in the word occur in developing countries (Vijayakumar, 2004). The highest suicide rate of Europe is reported in Eastern Europe (Lester, 2012). Sixty percent occur in Asia (Evidence, Team, & Project, 2006). According to the World Health Organization, about 40% of all suicides occurs in the world in China, India and Japan (Värnik, 2012). Suicide is considered the fifth cause of death in Iran (Saberi-Zafaghandi, Hajebi, Eskandarieh, & Ahmadzad-Asl, 2012). Previous suicide attempt is the most important predictor of death by suicide (Kataoka, Stein et al., 2007). Thus, it is also important to investigate suicide attempt cases. People who have had an earlier suicide attempt are 12 to 30% more likely to reattempt and the increased risk of death by suicide is higher in the first 1 to 3 years after their first suicide attempt (Cedereke, Monti, & Öjehagen, 2002; Vaiva et al., 2006). History of suicidal behavior is a risk factor for suicide reattempts (Malakouti et al., 2008). Around 40% of people died by suicide have at least one previous attempt (Cavanagh, Carson, Sharpe, & Lawrie, 2003). There is strong evidence that suicide is preventable (Ono, 2006). Preventing suicide is one of the central parts of the WHO’s operational program aimed to decrease suicide rate in countries by 10% in 2020 (World Health Organization, 2014). National suicide prevention strategy can be most effective by determining related risk factors, trying to mitigate these factors and using proper interventions. Proper access and accepting care can be effective in mitigating suicidal behavior risks (Cho et al., 2013). Ambivalence to treatment and repeated suicidal behavior are problems in people who have attempted suicide (Cedereke et al., 2002). Interventions after suicide attempt are therefore essential to prevention (Granboulan, Roudot-Thoraval, Lemerle, & Alvin, 2001). Follow-up after discharge from a hospital after a suicide attempt is one type of intervention and can include phone calls or other types of contact such as postal cards, email and text messages.

### 1.2 Aim

This article determines whether preventive interventions by phone or other vehicles (postal cards, email and case management) are effective in reducing reattempts after discharge. We will provide an overview of studies on suicide reattempts and discuss the effect of various methods of follow-up on the success of treatment in suicidal attempts.

## 2. Methods

### 2.1 Study Design

A narrative review was performed. Narrative reviews are the traditional approach mainly based on the experience and subjectivity of the author (Cipriani and Geddes, 2003).

### 2.2 Search Strategy

The search was done using keywords “suicide attempt”, “suicide prevention” and “follow-up” in PubMed, Google Scholar, PsycINFO and All EBM Reviews (OVID) with no time limit on gaining access to the published studies in English. To access published Iranian research, some Iranian databases including Iran Medex, Google, Scientific Information Database (SID) and the published information database of the country (Magiran) were searched with the same keywords in Persian language.

### 2.3 Inclusion & exclusion Criteria

We included all cross-sectional, longitudinal, cohort or case-control analytical designs, descriptive studies and clinical trials reporting on suicide interventions and follow-up services and published in peer-review journals. Only articles in English or Persian were included. Studies not related to follow-up after discharge was excluded.

### 2.4 Article Categorization

**Figure 1:**
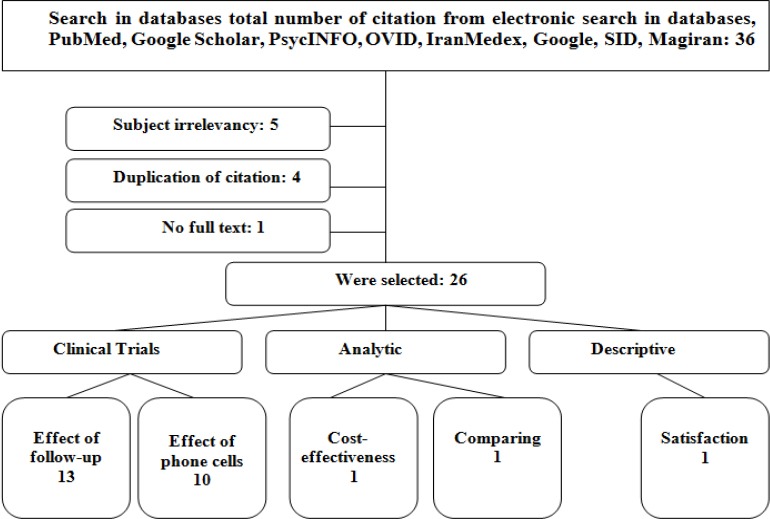
Study selection flow chart

From 36 total articles gathered in our search, 26 were selected for reading. Figure 1 presents a summary of study selection process.

## 3. Results

### 3.1 Literature Review

Research reported on articles in this review had been conducted from 1995 to 2014. We included 26 major types of studies for which the research question was clearly defined. These studies included research on suicide reattempts, the effect of follow-up calls on evaluating the success of treatment, treatment adherence and different aspects of the follow-up. The remaining articles which were not related to follow-up after discharge were outside the scope of this research.

### 3.2 The Recent Years

#### 3.2.1 Clinical Trials and Analytical Studies

From 25 cases discussing the effects of follow-ups after discharge including visits, mails, phone messages, postal cards, crisis lines and case management, 12 studies showed a significant decrease in suicidal behaviors. But one study reported no significant difference between suicides reattempts in an intervention group following up with postal cards and a control groups which only received standard treatment because some control participants were inadvertently exposed to the intervention, some intervention participants did not consent to get the intervention, and some intervention participants were not exposed to the intervention (Carter, Clover, Whyte, Dawson, & D’Este, 2005). Three studies from 10 intervention studies investigating the effect of phone calls/text message on suicide reattempts did not show any significant decrease in reattempts in the intervention groups (Berrouiguet et al., 2014; Vaiva et al., 2006; Mousavi, Zohreh, Maracy, Ebrahimi, & Sharbafchi, 2014). One article investigating the cost-effectiveness of phone follow-ups reported better results in the phone call groups than the cognitive behavior therapy group (Pil, Pauwels, Muijzers, Portzky, & Annemans, 2013). Another research comparing a group of suicide attempters with follow-up treatment to a group without it reported significant depression and anxiety in the control group in comparison with the intervention group. It also significantly showed more individuals in the control group planned their suicide reattempt (Granboulan et al., 2001). Table 1 presents a summary of reviewed RCTs in this research in recent years.

**Table 1:** Preventive interventions of suicide attempt in RCTs* studies

Authors	Findings
Mousavi SG, et al.(2014)	139 patients who have suicide attempt were studied and divided into a brief interventional control (BIC) and a treatment as usual (TAU) as control group. Phone follow-up program was performed systematically for the intervention group during 6 months after discharge. The control group received the routine treatment after discharge. No significant differences of suicide reattempt found between two groups (p=0.18).

Berrouiguet S, et al. (2014)	A multicenter randomized controlled study was conducted in 2014 on 530 patients. This study was to evaluate the impact of text messaging in reducing suicidal attempts and reduce the costs of interventions during a 6 and 13 months period. Participants included patients discharged from the emergency department or psychiatric unit. Although Intermediate analysis on the first 250 participants was not significant (p <0.5%), it will be expected to reach a results in the end of study by researchers. The final results have not yet been reported and published.

Cebrià AI, et al. (2013)	991 patients being hospitalized and discharged after committing suicide were studied and categorized into an intervention group (running phone management program) and a control group. Phone follow-up program was performed systematically for the intervention group after one week, 1-3-6-9 and 12 months after discharge. The control group received the routine treatment after discharge. Phone management program delayed the suicide attempt of the intervention group in comparison with the control group. Moreover, there was a lower rate of suicide reattempts in the intervention group.

Wei S, et al. (2013)	239 people who have the history of suicide were categorized in 3 groups: cognitive therapy group, phone intervention group and control group. Each group was separately checked 3-6 and 12 month later. After 12 months, a drop out of the phone intervention group was lower than the rest. There was 6.5% suicide reattempt in the control and cognitive therapy group and 1.2% in the phone intervention group. Patients accepted the phone intervention group more than the other two groups.

Marasinghe RB, et al. (2012)	68 participants were studied after committing suicide. Intervention including problem solving treatment, meditation, a short intervention for increasing social support and phone follow-up. The control group received the common treatment. Participants getting the intervention experienced a significant improvement in reducing suicidal thoughts and depression in comparison with the control group. The effect of this intervention on reducing suicide attempt was also presented.

Chen W-J, et al. (2012)	4765 of people committing suicide from 2006 to 2008 were monitored for 6 months through case management. Survival analysis results showed that the risk of suicide reattempt was significantly reduced for the intervention group in comparison with the control group. The proportional risk of suicide reattempt was 2.93 times more for the control group (confidence coefficient 3.47–2.47) and this risk proportion, according to Cox analysis, showed a more significant effect for men in comparison with women.

Hassanian-Moghaddam, H., et al. (2011)	2300 people admitting in Loqman Hakim hospital in Tehran because of suicide attempts were evaluated in two groups of intervention and control. Among 2113 final respondents, proportional risk of suicidal thought were reported as 0.31, with confidence distance of 0.38–0.22 and proportional suicide attempt reduction of 0.42 with confidence distance of 0.63–0.11.

Ho W-W, et al. (2011)	A study was conducted in 2011 targeting the suicide prevention program in Taiwan. During four years of study, calls with these lines were respectively 1328, 2625, 2795, 2989 and suicide rates were reported respectively 21.4, 20.1, 18.2, 17.8 among 100 000 people. Results of investigating 1076 people receiving and 197 people not receiving phone interventions showed that the probability of suicide attempts in the intervention group was decreased 2.08 times.

Asarnow JR, et al. (2011)	181 participants were studied. The intervention group received family support and phone call follow-ups after discharge while the control group received the common treatment. The acceptance rate of outpatient treatment was higher (92% vs. 76%, p=0.004) in the intervention group and results support the positive effect of intervention.

* Randomized controlled trial.

#### 3.2.2 Descriptive Study

Only a descriptive study found that investigating the satisfaction of people calling the national suicide prevention line concluded that participants were reported positively satisfied after calling that center (Coveney, Pollock, Armstrong, & Moore, 2012)(Table 2).

**Table 2:** Suicide attempt and follow-up services in a descriptive study

Authors	Findings
Coveney CM, et al. (2012)	This study discussed the satisfaction of the callers of the suicide prevention national phone line. Participants included 77.9% women and 22.1% men. Most callers were from 16-24 years old with 26.1%, 62.2% of participants were single and 79.1% lived with their families. More than 23% of the participants worked full-time and 20.1% were high school and university students. Results showed that 70% of the people, felt depressed and anxious before the call, while their depression and anxiety was significantly reduced after the call. More than 60% of the participants were reported to be positively satisfied after calling this center.

### 3.3 The Previous Years

Table 3 presents a summary of Preventive interventions and follow-up services in the previous years.

**Table 3:** Follow-up services of suicide attempt in RCTs* studies

Authrs	Findings
Fleischmann et al., 2008	1867 Suicide attempters in five culturally different sites (Campinas, Brazil; Chennai, India; Colombo, Sri Lanka; Karaj, Islamic Republic of Iran; and Yuncheng, China) received either treatment as usual, or treatment as usual and brief intervention and contact (BIC), which included patient education and follow-up. Findings showed Significantly fewer deaths from suicide in the BIC than in the treatment-as-usual group (P < 0.001).

Vaiva G, et al. (2006)	605 patients discharged after committing suicide were studied. Phone follow-ups with the patients were performed one and three months after discharge to evaluate the treatment success and acceptance in intervention groups. The control groups received the common treatment. Three groups had no significant difference in aforementioned factors. Participants who received follow-ups at the first month had less suicide reattempt (12% against 22% of the control group).

Carter GL, et al. (2005)	772 patients were discharged after committing suicide and targeting the effect of intervention using postal cards, were randomly divided into two groups of intervention and control. In their study, the intervention groups receiving postal cards against the group only receiving standard treatment were monitored for 12 months. Results showed no significant difference between the suicides reattempts of the intervention and control.

Cedereke M, et al. (2002)	216 patients were randomly divided into two groups. The intervention group received phone follow-up and the control groups did not. Interventions caused support and motivation for maintaining treatment. Follow-ups were received the first and the 12th month. Results showed the positive effect of phone follow-up among these patients.

## 4. Discussion

Studies have shown that short-contact interventions can have impact in reducing suicidal attempts (Milner, Carter, Pirkis, Robinson, & Spittal, 2015). Most psychiatric disorders including depression are chronic and recurrent able (Keller, Lavori, Rice, Coryell, & Hirschfeld, 1986). Thus accepting and maintaining medical treatment for these patients is usually difficult. Follow-up interventions enable more effective treatments (Khaleghparast et al., 2013; Vergouwen, Bakker, Katon, Verheij, & Koerselman, 2003). The risk for suicide attempt is high immediately after discharge (Angst, Stassen, Clayton, & Angst, 2002; Crawford, 2004; Vergouwen et al., 2003). In 1999, Geddes et al. showed that most suicide attempts occur during the first month after discharge with a sudden increase during the first week. This rate is more than a hundred times in general population. Thus it is essential to persist and maintain follow-up treatments (Geddes, 1999; Katon et al., 1996; Katon et al., 1995). Several studies have shown that follow-up support after discharge can significantly decrease the risk of suicide attempt (Wells et al., 2005).

In 2013, David et al discussed the significant effects of suicide follow-up interventions by follow-up calls with patients (Luxton, June, & Comtois, 2013). Randomized controlled trials on suicide reattempt prevention show less reattempt rates for intervention groups in comparison with control groups after receiving interventions like regular post mails, phone calls, and long-term follow-up treatments after discharge (Aoun, 1999; Asarnow et al., 2011; Cebrià et al., 2013; Cedereke et al., 2002; Gruat, Cottencin, Ducrocq, Duhem, & Vaiva, 2010; Marasinghe, Edirippulige, Kavanagh, Smith, & Jiffry, 2012; Vaiva et al., 2006; Wei et al., 2013). Most patients having attempted suicide suffer from psychiatric disorders and a significant number require outpatient psychiatric services (Sobolewski, Richey, Kowatch, & Grupp-Phelan, 2013). A survey during ten consecutive years showed that phone follow-ups have prevented 36% of suicide reattempts and thus saved treatment costs (Pil et al., 2013).

Previous research has shown that having a history of one suicide attempt can predict reattempts. But few studies show whether the characteristics of the people reattempted suicide is significantly different from their first attempt. Knowing the characteristics of these people helps predicting the risk of suicide reattempts. Studies have shown that 33% of people reattempting suicide possess characteristics different from their first attempt and 31% possess characteristics similar to their first, such that their feeling of loneliness, serious wish of death, one-hour or more planning for suicide attempt are significantly relevant to their suicide reattempt. Moreover their suicide method is the same from their first attempt to the last (Miranda, Jaegere, Restifo, & Shaffer, 2014). Reviewing intervention therapies showed that patients accepting treatment are less probable to reattempt in comparison with patients avoiding treatment. Only 25-50% of patients pay attention to doctors` and nurses` outpatient psychiatric services at the first month. Evidence show follow-ups after discharge from psychiatric hospitals and emergency units are effective treatments for decreasing the risk of suicide reattempts. Follow-up support can be used in many different ways (phone calls, crisis cards, mails, postal cards and the like.) for patients at risk of suicide reattempts (Vaiva et al., 2006). In 2012, Luxton et al showed that most patients prefer emails to mails for follow-up therapies (Luxton et al., 2013). In 2010, Chen et al reported that follow-up intervention using test messages and cellular phones were a useful and acceptable follow-up method for 80% of patients (H. Chen, Mishara, & Liu, 2010).

Research shows that postal cards are less important and cognitive therapy is more effective that common treatments in decreasing suicide reattempt (Yip, 2011). It seems for a suicide reattempt preventive plan to succeed; social support and developing follow-up programs decrease suicidal behaviors. Another research about patients attempting to suicide in 5 countries, Brazil, Holland, Sri Lanka, Iran and China, categorized participants in two groups: a control group receiving common treatment and an intervention group receiving common treatment, also a one-hour training in emergency room and phone follow-ups during weeks and months after discharge. This study showed significance decrease in suicide reattempts in the intervention group, which showes consistent, periodical follow-up through contacting the patient (Yip 2011). A study in China aimed to investigate the effect of interventions on reattempts, drop outs in cognitive therapy and control groups were more than phone intervention groups which showes phone intervention groups had better acceptance in comparison with the former (Wei et al., 2013).

A study in Italy about follow-up after discharge on the elderly attempting to suicide shows a decrease in reattempts (Wood, Bellis, Nurse, & Sirotkin, n.d.). In summation, over viewing the research showes that most intervention studies investigating the effectiveness of phone follow-up programs have reported similar results, such that after 12 months, drop outs, also suicide reattempts are less probable in phone intervention groups that other groups which shows accepting treatment by the people in those groups (Wei et al., 2013). In 2013, Affect Disord announced that intervention groups (phone management program) faces less and more delayed suicide attempts (Cebrià et al., 2013). In a study aimed to investigate the effect of Brief Mobile Treatment (BMT) to prevent suicide, participants receiving intervention experienced a significant improvement in suicide and depression in comparison with the group receiving common care (Marasinghe et al., 2012).

Research shows that regular phone calls are effective in mitigating the risk of suicide reattempts, such that 78.8% of patients receiving phone follow-ups expressed it as useful, 40.4% as effect on their lives and 29.4% considered it positively on preventing suicide reattempts. In summation, 94.5% of patients believed that a phone call is the most proper method to contact them (Gruat et al., 2010). A study investigating the effect of frequent phone calls on accepting treatment, repeating suicidal behavior and mental health, one year after committing suicide, found positive effects of phone follow-ups, performed in an intervention group, 1 and 12 months after discharge (Cedereke et al., 2002).

Among the intervention studies above which investigated the effectiveness of phone follow-up program, only one conducted on 605 patients, 13 of which discharged from the emergency unit in the north of France, did not show any significant difference during a 13 month follow-up between the intervention group (phone follow-up, one month and three months after discharge) and the control group (common treatment) according to suicide reattempts, The deaths caused by suicide and drop outs (Vaiva et al., 2006). This can be related to low acceptance of follow-up treatments by these patients. Moreover, another clinical trial which evaluated the follow-up operations by sending postal cards against a control group which only received standard treatment, reported no significant difference between reattempts of the control and intervention groups (Carter et al., 2005). Although results of the research conducted in 2010 in Iran aimed to investigate the follow-up effect of postal cards on suicide reattempts, reported proportional risk reduction of suicidal thoughts and reattempts (Hassanian-Moghaddam, Sarjami, Kolahi, & Carter, 2011).

Results of services provided by suicide prevention crisis lines, contacting counseling lines and using case management also show a decrease in suicide reattempt of the intervention group (W.-J. Chen et al., 2012; Ho et al., 2011). Moreover, research shows that more than 60% of the callers of the national suicide prevention phone line feel more positively satisfied after receiving phone counseling services. In summation, research shows that providing comprehensive aids, social support and follow-up after discharge can significantly prevent suicide reattempts.

## 5. Conclusion

Among patients who have attempted suicide and discharged from the emergency or psychiatric units, 25% reattempts suicide and this shows the need of follow-ups for these patients. Most studies show the positive effect of follow-up after discharge, particularly phone follow-up, on preventing suicide reattempts. It is suggested to perform follow-ups 6-12 months after discharge by medical team. Support follow-up systems and strong interaction through phone calls help for patient having attempted suicide. Patient satisfaction is effective in the treatment quality and must be considered for phone call follow-ups after suicide attempts. There is also a need for more research about defining consequences, measuring dependent variables, effects related to gender and high risk groups. It is required to develop and evaluate new approaches and use more diverse populations to compare current routine treatments more accurately with findings of the new research.

It is true that frequent phone call follow-ups mitigate suicidal behaviors; however more randomized controlled trials (RCT) are required to determine what factors of follow-up are more effective than others.
